# LNVA for treating secondary lymphedema: A systematic review

**DOI:** 10.1016/j.jpra.2026.07.035

**Published:** 2026-08-03

**Authors:** Caroline Lilja, Wahida Chakari, Jens Ahm Sørensen, Jørn Bo Thomsen

**Affiliations:** a Research Unit for Plastic Surgery, Odense University Hospital, J. B. Winsløws Vej 4 5000 Odense C, Region of Southern Denmark, Denmark; b Department of Clinical Research, University of Southern Denmark, Campusvej 55 5230 Odense M, Region of Southern Denmark, Denmark; c The Department of Plastic Surgery, Odense University Hospital, J. B. Winsløws Vej 4 5000 Odense C, Region of Southern Denmark, Denmark

**Keywords:** Lymphedema, Microsurgical reconstruction, Lymph-node to vein anastomosis, LNVA

## Abstract

Secondary lymphedema is a frequent long-term complication following cancer treatment. Although several surgical options exist, lymph node-to-vein anastomosis (LNVA) has recently gained renewed attention as a potential treatment for secondary lymphedema. This systematic review aimed to evaluate the current evidence regarding the efficacy of LNVA in the treatment of secondary lymphedema. A literature search was conducted on November 10, 2025, in Embase, MEDLINE, the Cochrane Library, and Google Scholar. ClinicalTrials.gov and the International Clinical Trials Registry Platform were also screened for ongoing studies. Studies reporting outcomes after LNVA in patients with secondary lymphedema were included. Two independent reviewers performed study screening and data extraction. Risk of bias was assessed using ROBINS-I and the JBI Critical Appraisal Checklist for Case Series, and the certainty of evidence was evaluated using GRADE. Three studies met the inclusion criteria: two retrospective cohort studies and one case series. Across studies, LNVA was associated with modest reductions in limb circumference and partial symptom relief in some patients. However, all studies demonstrated serious or high risk of bias, and the overall certainty of evidence was rated as very low. Current evidence supporting LNVA for secondary lymphedema is limited and of low methodological quality. Well-designed prospective studies are required to determine the true clinical value of LNVA.

## Introduction

Secondary lymphedema continues to be a significant long-term sequela following cancer treatments, affecting a wide range of patients (estimated between 9% and 67%).^1^ While surgical treatments such as lymphovenous anastomosis (LVA), vascularized lymph node transplants, and liposuction can, in some cases, relieve symptoms amongst patients, there is still a need for an effective treatment of lymphedema.^2^

Therefore, surgeons continue to develop new techniques and procedures to improve treatment strategies and help patients manage their symptoms and improve their quality of life. One of these surgeries is lymph node-to-vein anastomosis (LNVA), which has shown promising results.^1^^,^^3^^,^^4^ It was first described by Calnan et al., who performed the surgery on dogs in 1967, shortly followed by Nielubowicz and Olszewski in 1968, who performed the surgery on four patients with secondary lymphedema of the lower limbs, resulting in a circumference reduction of 3–10 cm.^5^^,^^6^ Despite this, the procedure never gained widespread acceptance and was not further used for the treatment of lymphedema, possibly due to inconsistent results. However, with the advent of more refined equipment and robot-assisted microsurgery, enabling accurate microsurgery on smaller structures, a new hope for the procedure has arisen.^3^ Combined with more refined surgical techniques, using a puncture of the lymph node capsule rather than a cut surface of the lymph node, the nodal architecture may be better preserved, potentially increasing the procedure’s effectiveness in improving lymphedema symptoms.^3^^,^^5^^,^^7^^,^^8^

The surgery itself involves identifying functional lymph nodes proximal to the lymphedematous area and, by making a small opening in the lymph node capsule, anastomosing a nearby vein directly to it in an attempt to alleviate the compromised lymphatic system and improve lymph drainage.^1^^,^^3^ It is hypothesized that direct drainage from the lymph node may improve symptoms for pitting lymphedema, compared to other microsurgical procedures.^1^^,^^3^^,^^4^ And while a single LVA drains the small area of that specific lymph vessel, the inguinal LNVA can drain numerous collectors from several lymph nodes, possibly offering an effective alternative to LVA.^3^ Although LNVA has been described in clinical discussions as an advanced extension of conventional LVA, there seems to be little knowledge on patient selection and surgical outcomes, and to our knowledge, no systematic review of the current evidence has been conducted. Therefore, the aim of this systematic review was to compile existing knowledge on LNVA to evaluate patient selection and the procedure's efficacy in treating secondary lymphedema.

## Method, search strategy, and eligibility criteria

A literature search was conducted on November 10, 2025, in the following four databases: Embase, Medline, Cochrane Library, and Google Scholar. In addition, ClinicalTrials.gov and the International Clinical Trials Registry Platform (ICTRP) were searched for any ongoing trials. No filter function nor time limit was applied. The search query included keywords such as: “lymph node to vein anastomosis”, “LNVA”, and “lymphedema” or “lymphatic disease”, see appendix for full search string.

The research question was formulated according to the PICO framework, where P represents the study population, I the intervention, C the comparator, and O the outcome of interest.^9^ The PICO model for this review was:

P: Patients with secondary lymphedema

I: Lymph node to vein anastomosis

C: No intervention, conservative treatment, other surgical intervention, or no comparison group.

O: Limb volume, circumference measurements, quality-of-life measured with validated questionnaires, degree of fibrosis, tissue water content, or L-Dex score measured with bioimpedance, or any other outcome measure for evaluating the effect.

Our inclusion criteria included articles on patients with secondary lymphedema treated with LNVA, where the effect has been evaluated using an objective outcome measure. See Table 1 for full criteria. The reference lists of full-text articles were also screened for relevant articles.

**Table 1:** The inclusion- and exclusion criteria for this systematic review.

Inclusion criteria	Exclusion criteria
• Secondary lymphedema	• Conference abstract or comments
• Treated with lymph node to vein anastomosis	• Animal studies
• Outcome measure of any kind to evaluate the effect	• Written in a language other than English, Danish, Norwegian, or Swedish
• Randomized controlled trials, prospective- and retrospective studies, case-series and case-reports	

The article was written following the PRISMA guidelines.^10^

## Data extraction

Two independent reviewers screened titles and abstracts using Covidence (Melbourne, Australia) software. The same reviewers subsequently performed full-text screening. Disagreements were resolved through discussion between the two reviewers, with a third reviewer available for discussion if consensus could not be reached.

For the included studies, study characteristics were collected in an Excel spreadsheet, including the study design, year of publication, country of origin, number of patients, type of comparison group, lymphedema duration, lymphedema classification, surgical description, surgical duration, outcome measure, follow-up duration, post-operative management, and side effects.

Risk of Bias and Quality Assessment

The risk of bias of the included studies was assessed using the ROBINS-I for non-randomized controlled trials, and the JBI Critical Appraisal Checklist for Case Series.^11^^,^^12^ The evidence was assessed using GRADE.^13^ As the included studies did not provide sufficient data for a combined analysis, a meta-analysis was not performed.

## Statistical analysis

Due to the limited number of relevant studies, no meta-analysis was performed. The findings are therefore presented narratively, summarizing the key trends.

## Results

**Fig. 1:**
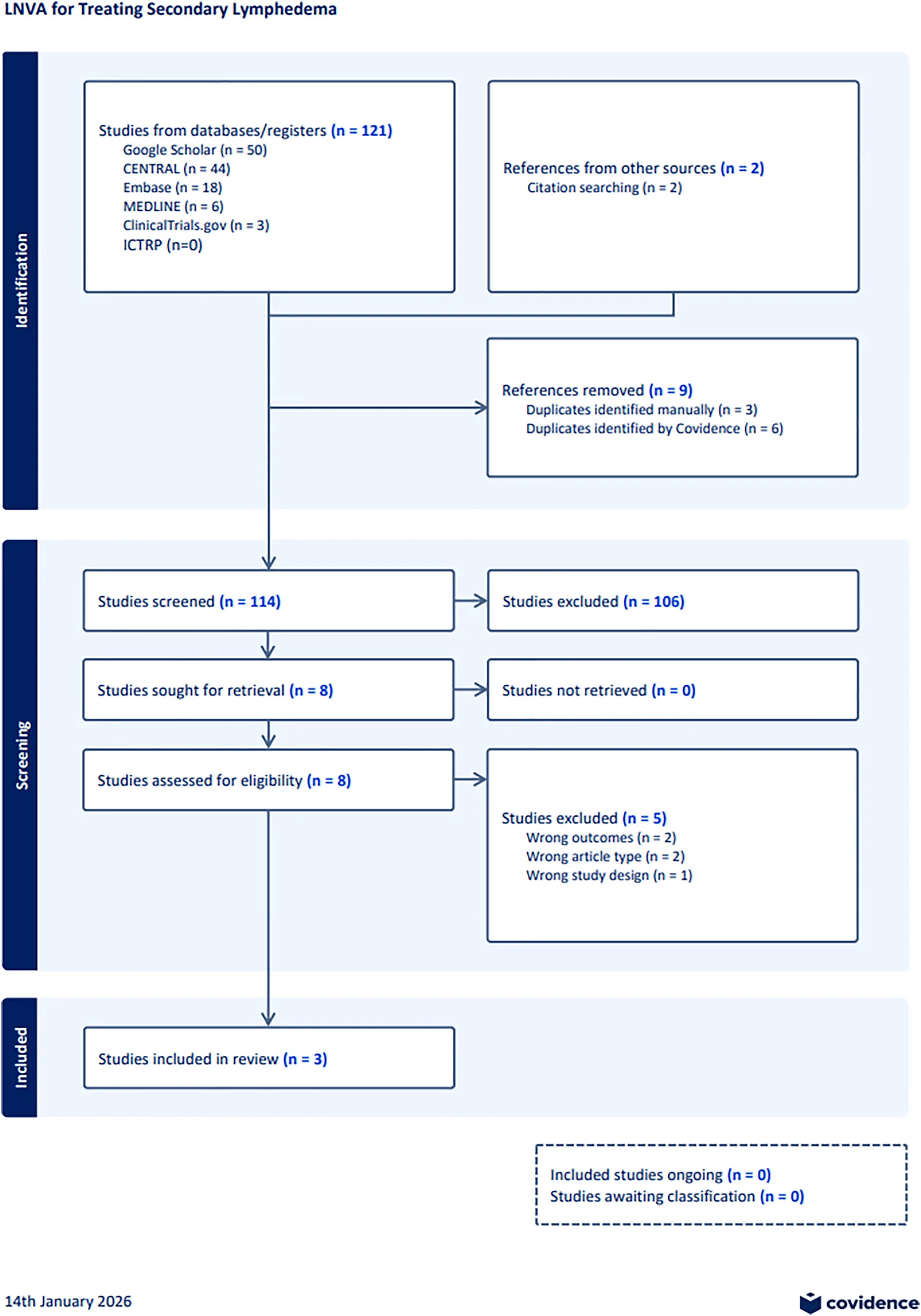
PRISMA flowchart illustrating the study selection process for this systematic review. A total of 114 records were identified after duplicates were removed and screened by title and abstract. Eight studies underwent full-text review; three met the eligibility criteria and were included in the qualitative synthesis.

The literature search yielded 114 studies after removing duplicates, of which eight were included for full-text screening.^1^^,^^3^, ^4^, ^5^^,^^7^^,^^8^^,^^14^^,^^15^ After thorough evaluation, only three studies fulfilled our eligibility criteria, one from Korea and two from Poland.^1^^,^^5^^,^^8^ No ongoing studies were identified. See Fig. 1 for the PRISMA flow-chart of the screening process, and Table 2 for full study characteristics. Across studies, LNVA was associated with modest reductions in limb circumference and pain in a subset of patients; however, outcomes were inconsistently reported and pooled across surgical techniques.^1^^,^^5^^,^^8^

**Table 2:** Overview of the included studies and their study characteristics. LNVA: Lymph node-to-vein anastomosis, LVA: Lymphovenous Anastomosis.

Authors	Year of plublication	Country of origin	Study Design	Number of patients	Intervention	Comparison	Lymphedema classification	Outcome measures	Follow-up duration	Post-operative management	Adverse events
Pak et al.	2021	Korea	Retrospective cohort study	160	LNVA + LVA	LVA	ISL(Stage II: 105,Stage III: 55)	Limb circumference, Pinch test, body composition	23.3 months(13–48)	Immediate compression,mobilization post-operative day 1	n/a
Olszewski W. L.	2013	Poland	Retrospective cohort study	1176	Lymphodeno-venous shunt, or lymhpatico-venous shunt	None	Author-defined classification	Limb circumference, flexing angle in ankle and knee joint, McGill Pain Questionnaire	5–40 years (results after 5 years presented)	Light massage to the limb.	None
Nielubowics J., Olszewski W. L	1968	Poland	Case series	4	LNVA	None	Author-defined classification	Leg circumference, movement of joint, pain evaluation, evaluation of oedema presence	1–9 months	n/a	n/a

**Fig. 2:**
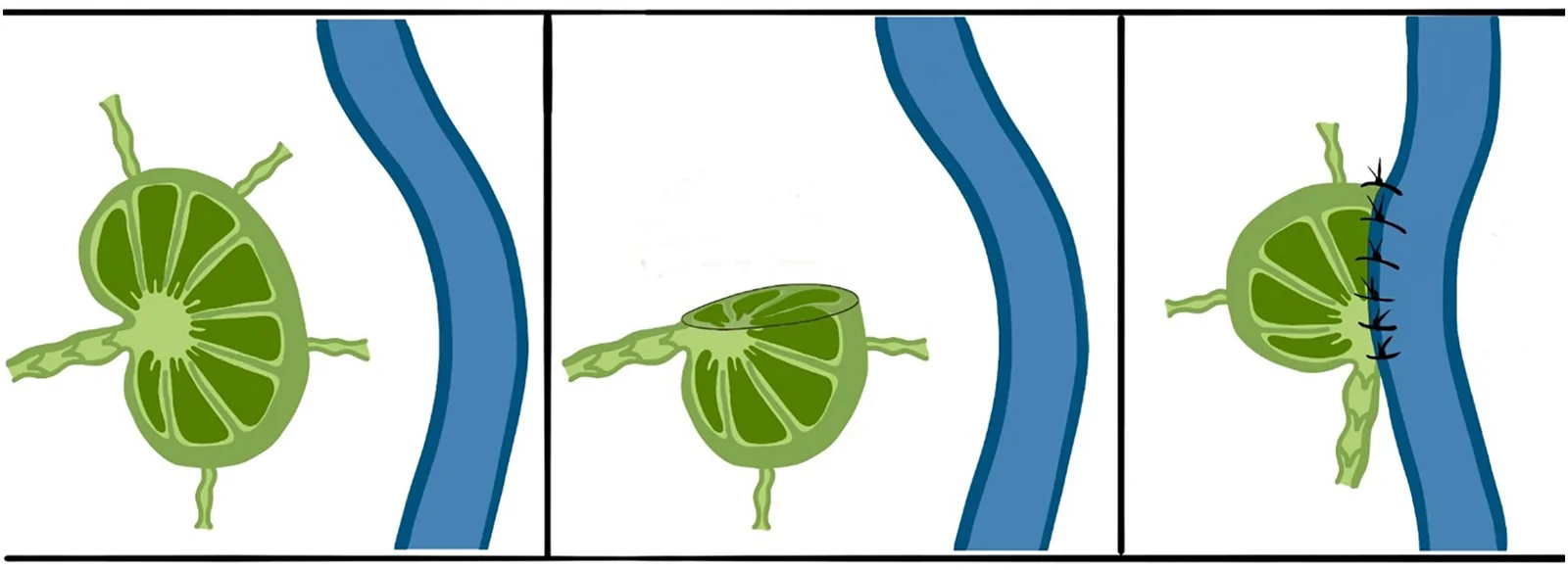
Illustration of the surgical technique, by Nielubowicz J. and Olszewski W. from 1968, with transection of the lymph node through an equatorial plane before anastomosing onto the nearby vein.

The first study, by Nielubowicz J. and Olszewski W. from 1968, describes the first experiences with anastomosing a lymph node to a nearby vein in 4 patients.^5^ The four patients all had developed lymphedema following gynecological cancer, and all received anastomosis between an inguinal lymph node and the saphenous vein or femoral vein. The inguinal lymph node near the saphenofemoral junction was identified by blue staining after patent blue injection into the medial thigh. The node was mobilized and transected at the equatorial plane. A nearby vein was then dissected and incised to create an anastomosis between the node capsule and the vein wall, see Fig. 2. The first patient died after 9 months of follow-up due to metastases and cachexia, which enabled histological examination of the anastomosed lymph node, showing four lymphatic channels running into the opening of the vein. After 7 months, her leg circumference had decreased by 10 cm. The remaining two patients had circumference reductions ranging from 3 to 7 cm. In patient three, edema was reported as absent at rest but occurred during walking.^5^

Thirty-five years later, Olszewski W. published his experiences from 45 years of performing shunt treatment for lymphedema, making it the second included study of this review.^8^ The article included 1343 patients, of whom 983 were females, and 357 were males, treated between 1966 and 2011. Patients were grouped by lymphedema etiology (postinflammatory/posttraumatic, postsurgical, idiopathic, and hyperplastic), and clinical staging was determined by clinical evaluation of edema, skin keratosis, and fibrosis, correlated with lymphangiographic images, according to the author’s staging system.^8^ Patients received either a lymph node-saphenous vein (LNSV) or a lymphatic vessel-saphenous vein (LVSV) anastomosis in the groin, depending on the presence of inguinal lymph nodes. Six hundred and fifty patients out of the 1343 received LNSV surgery. During surgery, the inguinal lymph node was transected transversely along its long axis to allow anastomosis of the capsule to a nearby vein.^8^

Patients were assessed every six months using limb circumference measurements, ankle and knee joint flexion, and pain was evaluated with the McGill Pain Questionnaire. Improvement was defined as a composite score of 2–4 points, based on predefined thresholds for circumference reduction, joint mobility, and symptom relief. Outcomes were reported for the pooled cohort, preventing comparison between lymph node–saphenous vein (LNSV) and lymphatic vessel-saphenous vein (LVSV) procedures. Improvement was observed in 29–42% of patients with postinflammatory lymphedema, accompanied by significant reductions in limb circumference at multiple levels (p < 0.05), improved joint mobility, and reduced pain. Among postsurgical lymphedema patients, improvement was reported in 49–80% of those with stage I-II disease. Improvement rates were 33–41% in stage I idiopathic lymphedema and 0–14% in stage II, while 82–100% of patients with hyperplastic lymphedema improved. See Table 3 for all results. Long-term follow-up of 218 patients (10–40 years) showed sustained improvement in 19–50% of cases, with no reported disease progression.^8^

**Table 3:** Overview of Olszewzki W. L. results, with data prior to surgery and after five years of follow-up for patients experiencing an lymphedema improvement after undergoing either lymph node-saphenous vein (LNSV) anastomosis or lymphatic vessel-saphenous vein (LVSV) anastomosis. Data is presented as mean with its respective standard deviation.

	Outcome Measurements					
Lymphedema group	Circumference Ratio			Joint flexion		Pain
	Mid-foot	Mid-calf	Mid-thigh	Ankle	Knee	
Post-imflammatory lymphedema	1.5 ± 0.3 to 1.3 ± 0.4 (p < 0.05)	2.8 ± 1.4 to 2.5 ± 1.4 (p < 0.05)	1.8 ± 0.8 to 1.6 ± 0.8 (p < 0.05)	25° ± 10° to 35° ± 10° (p < 0.05)	60° ± 30° to 90° ± 20° (p < 0.05)	80% changed from heavy to dull. Severity decreased from 1–5 to 1–2.
Postsurgical lymphedema	1.3 ± 0.5 to 1.1 ± 0.5 (p < 0.05)	1.8 ± 1.2 to 1.6 ± 0.8 (p < 0.05)	2.0 ± 0.6 to 1.7 ± 0.8 (p < 0.05)	35° ± 15° to 45° ± 10° (p < 0.05)	70° ± 20° to 90° ± 20° (p < 0.05)	60% changed from heavy to sore. Severity decreased from 1–5 to 1–2.
Idiopathic lymphedema	1.3 ± 0.5 to 1.2 ± 0.4 (p < 0.05)	1.8 ± 0.4 to 1.6 ± 0.8 (p < 0.05)	n/a	n/a	80° ± 30° to 90° ± 20° (p < 0.05)	80% changed from heavy to dull. Severity decreased from 1–5 to 1–2.
Hyerplastic lymphedema	1.6 ± 0.5 to 1.1 ± 0.5 (p < 0.05)	2.2 ± 0.3 to 1.8 ± 0.9 (p < 0.05)	2.4 ± 0.6 to 2.1 ± 0.5 (p < 0.05)	“not restricted”	“not restricted”	n/a

The third included study, published in 2021, describes a retrospective cohort study of 160 patients with lower limb lymphedema who were either undergoing LNVA and LVA surgery or only LVA as a comparison.^1^ The LNVA and LVA group consisted of 97 lower limbs (60 International Lymphedema Classifications (ISL) stage II and 37 ISL stage III), and the control group consisted of 63 lower limbs (45 stage II and 18 stage III). If an inguinal node dissection was performed, the patient was excluded. All patients underwent pre- and post-operative measurements at 12 months of follow-up, including tape circumference at six levels to estimate limb volume, a pinch test of the suprapubic skin, and body composition measured by Bioimpedance.

The patients consisted of 137 women and 23 males with an average age of 62.5 (29-75), who had developed lymphedema secondary to uterine cancer (n = 116), ovarian cancer (n = 19), other malignancies (n = 20), or other reasons not classified (n = 5). One hundred and seventeen out of 160 patients had undergone radiation therapy. They were followed for a mean period of 23.3 months (13-48).

**Fig. 3:**
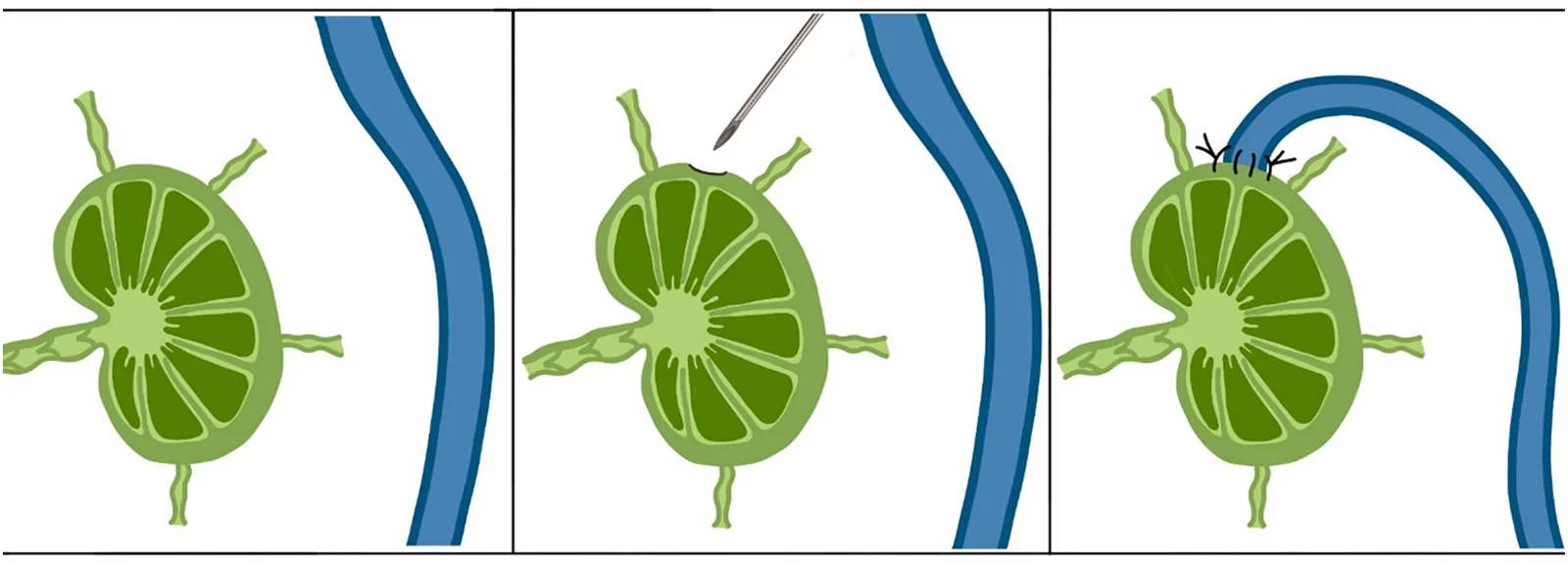
Illustration of the surgical technique, by Pak CS et al.​, using a needle to puncture the lymph node prior to anastomosing onto a nearby vein.

To plan the LNVA, lymphoscintigraphies and magnetic resonance lymphangiograms were performed to evaluate the size and location of the lymph nodes. As for the LVA, indocyanine green lymphography (ICG-L) combined with high-frequency ultrasound was used to identify functioning lymphatics. Perioperatively, filtered technetium-99 (Tc-99) was injected into the first and second web spaces of the foot and the lower abdomen on the affected side to identify inguinal lymph nodes using a gamma probe. During the dissection of the lymph nodes, any superficial veins were secured. And once the lymph node was identified, it was punctured with a 16-gauge needle and dilated with a dilatator. The anastomosis was then performed using a 10–0 nylon suture between the punctured lymph node and a nearby vein, see Fig. 3.

A significant circumference reduction of 2.6 ± 2.3 cm was observed in the LNVA+LVA group compared to 2.2 ± 2.1 cm in the LVA group for patients with lymphedema stage II, and a decrease of 2.2 ± 2.3 cm for LNVA-LVA compared to 1.6 ± 2.1 cm for the LVA group with lymphedema stage III. A significant reduction of the suprapubic pinch was also observed. As for the extracellular fluid within the affected leg, a significant improvement was also seen in the LNVA+LVA group compared to the LVA group (reduction of 1.94 versus 0.91, p < 0.05).^1^

## Risk of bias and quality assessment

The case series by Nielubowicz J. and Olszewski W. L. was judged to be at high risk of bias due to a small sample size, the lack of explicit inclusion criteria, and unclear consecutive or complete case inclusion criteria. In addition, reporting of outcomes and follow-up duration was inconsistent.^5^ See Table 4, Table 5.

**Table 4:** Risk of bias (ROB) assessment and evaluation of the quality of the evidence for the included studies.

Study	Design	ROB Tool used	Overall Risk of Bias	Quality of the Evidence (GRADE)
Pak et al.	Retrospective cohort study	ROBINS-I	Serious	⊕◯◯◯ Very low
Olszewski W. L.	Retrospective cohort study	ROBINS-I	Serious	⊕◯◯◯ Very low
Nielubowics J., Olszewski W. L	Case series	JBI Case Series	High	⊕◯◯◯ Very low

**Table 5:** Risk of bias (ROB) assessment and evaluation of the quality of the evidence for the included studies of non-randomised trials, using ROBINS-I. Domains: D1: Bias due to confounding, D2: Bias in classification of interventions, D3: Bias due to selection of participants, D4: Bias due to missing data, D5: Bias in measurement of the outcome, D6: Bias in the selection of the reported results. Yellow circle with a minus in the center: Moderate; Red circle with an “X” in the center: Serious; Red circle with an exclamation mark in the center: Critical.

	**D1**	**D2**	**D3**	**D4**	**D5**	**D6**	**Overall**
Pak et al.							
Olszewski W. L.							

Using the ROBINS-I tool, the Olszewski W. L. study was judged to be at serious risk of bias.^8^ Although two surgical techniques were employed, results were reported in pooled analyses, and allocation to intervention type was non-random and based on anatomical considerations. The lack of a comparison group results in a serious risk of bias. Additional concerns include incomplete follow-up, lack of blinded outcome assessment, and limited reporting of results by surgical type, despite the authors stating that there were no differences in techniques or patient selection.

Pak et al. presented a study evaluating a refined surgical technique, though its retrospective design imposes inherent limitations.^1^ The intervention and comparison groups were clearly defined; however, patients selected for LNVA surgery typically had a less compromised lymphatic system, as demonstrated by their ability to drain into the inguinal lymph nodes, whereas those without visualization were assigned to the comparator group. This allocation reduces the likelihood of misclassification but reinforces selection bias. As a non-randomized, retrospective comparison based on functional criteria, the study is susceptible to confounding by indication. Additional concerns include risk of bias from patient selection, measurement, and reporting, compounded by differences in follow-up duration and the absence of information regarding missing data or loss to follow-up. Although outcomes were assessed consistently and transparently, the non-randomized design and incomplete follow-up information limit causal interpretation and an overall evaluation of the serious risk of bias.

The evidence was assessed using GRADE. As all included studies were observational, the level of evidence was low. The evidence was further downgraded due to risk of bias, selection bias, and imprecision related to small sample sizes. The overall certainty of the evidence was therefore assessed as very low.

## Discussion

This systematic review shows a small number of articles investigating the efficacy of LNVA for treating lymphedema. Although there is a new wave of potential for the intervention, there is very little evidence to support effective treatment at this time.^1^^,^^5^^,^^8^

Early reports demonstrated a modest reduction in limb circumference and some pain relief following LNVA; however, these studies were retrospective and employed invasive techniques that may have compromised lymph node integrity.^5^^,^^8^ More recent data suggest that refined LNVA techniques, combined with LVA, may lead to modest improvements in limb volume and extracellular fluid, especially in patients with lower-stage disease.^1^ Nevertheless, these findings are confounded by selection bias, as patients eligible for LNVA generally had better-preserved lymphatic function at baseline.^1^

The surgical technique has evolved since the original idea and theory of creating an anastomosis between a lymph node and a vein was introduced.^1^^,^^4^ The newer technique is likely superior at maintaining and preserving the lymph node's internal structure without disrupting its function.^1^ However, current evidence cannot prove that one technique is superior to another.^1^^,^^5^^,^^8^ In contrast, performing a transverse section through the entire lymph node represents a more invasive surgical approach, which may damage the lymph node and likely sacrifices some of its efferent vessels.^5^^,^^8^ Current trends, therefore, theoretically favor a more gentle surgical technique.^1^^,^^5^^,^^8^

In addition to improvements in surgical technique, likely facilitated by advances in technology and the development of finer microsurgical equipment, LNVA may have new potential for effective treatment of secondary lymphedema.^4^^,^^16^^,^^17^ The introduction of robot-assisted microsurgery further enhances the ability to perform more refined microsurgery. Some even argue it improves precision and flexibility, reduces fatigue and tissue damage, and makes complex tasks more consistent.^4^^,^^16^^,^^17^ All these new aspects create a growing potential for LNVA surgery in treating lymphedema.

When considering the newer technique described by Pak et al., no firm conclusions regarding its effect can be drawn from the available evidence, due to differences in patient selection between the intervention and control groups.^1^ Patients whose inguinal lymph nodes could drain and absorb Tc-99 injected at the foot are likely to have milder lymphedema and a better-preserved lymphatic system than patients whose lymph nodes could not be identified and were therefore placed in the control group.^1^ As a result, the intervention group is favored from the beginning, as these patients start in a better clinical condition. To determine the true effect of LNVA, a study is needed in which all patients are eligible for LNVA surgery, ideally a randomized controlled trial, allocating patients to either the treatment or a control group.^18^ A similar issue is seen in the study by Olszewski W.L., where patients without identifiable inguinal lymph nodes underwent LVA instead, and the results were reported together. This pooling of outcomes makes it impossible to clearly assess the intervention's actual effect.^8^

Pak et al. nevertheless support the hypothesis that patients with lower-stage lymphedema may benefit more from surgical reconstructive treatment than those with more advanced disease, as both treatment groups showed greater circumference reduction in stage II compared with stage III lymphedema.^1^ These findings also suggest that although physiological reconstructive techniques are primarily intended for fluid-dominant lymphedema, they may still reduce the fluid component in more advanced, fibrotic stage III disease. This observation opens the discussion on combined lymphatic treatment strategies, such as the addition of liposuction. However, the true effect of LNVA should first be clearly established before further exploration of combined approaches.

As for the eight articles included for full-text screening, three research groups were repeated and also represent those of the included studies, presenting experiences from Poland, Korea, and the United States. Papers were excluded either because no objective outcomes were presented for patient assessment or because of an incorrect study design.

Accurate evaluation of lymphedema is essential for establishing the diagnosis and guiding treatment.^19^ An initial assessment based on clinical evaluation of pitting using the pitting test, a detailed patient history focusing on symptom burden and daily management of swelling, and assessment of the response to conservative therapy. Medical history should clarify whether the lymphedema is secondary or primary and classify the disease level using the ISL.^4^^,^^19^ ICG-L is used to assess lymphatic function and identify lymphatic leakage, visualized as areas of dermal backflow with a stardust pattern.^20^^,^^21^

As lymph nodes are thought to play a crucial role in the LNVA procedure, the presence of functional lymph nodes may be important for improving lymphatic fluid drainage from the affected lymphosome.^3^ Even though the team from Pak et al. used magnetic resonance imaging (MRI) for evaluation of the inguinal lymph nodes in the included study, it is both time-consuming and cost-consuming to perform on patients.^1^ Alshomer et al. later evaluated the use of high-frequency ultrasound to assess functional inguinal lymph nodes and demonstrated that this method is effective in identifying functional lymph nodes in the groin.^7^ The identified lymph nodes are assessed based on shape, morphology, and vascular pattern using color Doppler and are subsequently classified as optimal, suboptimal, or not visualized.^7^ Once functional and anatomically suitable lymph nodes are identified, the patient is considered a candidate for LNVA surgery based on today's knowledge.^3^^,^^22^ Preoperative marking should include the targeted lymph node and vein identified by high-frequency ultrasound, and the planned incision site.^3^^,^^22^ After identification, the lymph node and vein are carefully prepared as described by Chen et al., with partial clearance of the lymph node capsule while preserving afferent and efferent lymphatic vessels and the hilar region.^4^ A vein of sufficient length to allow a tension-free anastomosis is selected and clamped before being mobilized toward the lymph node. The anastomosis may be performed using either a conventional microsurgical or a robot-assisted approach, depending on the surgeon’s preference. Following this, immediate compression therapy is recommended; however, no evidence is available regarding the optimal postoperative regimen.^1^^,^^5^^,^^8^

The lymphatic system operates as a low-pressure network (0–5 mmHg), whereas the venous system, particularly in upright positions, can exhibit substantially higher pressures, often exceeding 50 mmHg in dependent regions.^23^ Lymphatic capillary pressures are typically in the range of 4–10 mmHg, meaning that lymphatic drainage into the venous circulation occurs against an adverse pressure gradient and depends on active lymphatic pumping, osmotic pressure, and favorable local hemodynamics.^24^^,^^25^ Importantly, lymphatic pressure gradually increases along the proximal course of the system due to intrinsic contractility and cumulative flow, resulting in higher pressures in collecting lymphatics and near central lymphatic trunks compared to distal capillaries.^24^ As a result, the pressure gradient between the lymphatic and venous systems is relatively smaller at more proximal anatomical sites, such as the inguinal region, where lymph nodes and larger collectors are present, than in the distal extremities.^24^^,^^25^ This has direct surgical relevance in LVA and LNVA, as distal sites often exhibit a larger unfavorable pressure gradient, while more proximal locations may provide more favorable conditions for lymphatic outflow into the venous system.^24^, ^25^, ^26^, ^27^ On the other hand, if the irreversible pathological changes associated with lymphedema originate proximally and gradually extend distally, characterized by destruction of endothelial and smooth muscle cells leading to vessel occlusion, the more distal regions may be more favorable, as the lymphatic pump function may remain intact and thereby better support LVA performed at distal sites than LNVA at proximal sites.^26^ A combined approach, as described by Pak et al., may therefore be optimal; however, the current evidence is limited, and this question remains inconclusive.^1^ Further research is needed to fully evaluate the effects of LNVA and its potential benefits when combined with distal LVA.

Venous backflow into lymphatic channels has been described in LVA as venous-lymphatic reflux, where venous blood enters the lymphatic lumen after anastomosis formation. This phenomenon has been associated with reduced long-term patency of LVA and possible retrograde pressure into the lymph vessels.^28^^,^^29^ However, its true clinical impact is still not fully understood, and direct studies specifically quantifying venous backflow in LNVA are currently lacking.^30^ The use of a lymph node between the lymph system and venous system could, however, theoretically reduce the risk of venous backflow, as seen in LVA surgery.

## Strengths and limitations

One of the main strengths of this study is its systematic methodology, which included a comprehensive search across four databases and two trial registries. A key limitation is the lack of clinical studies in LNVA, leading to the inclusion of only three studies. However, this can also be a strength, as it provides transparency for clinicians and researchers in the field of lymphedema, highlighting the current lack of evidence supporting patient selection and the effectiveness of the surgery. Furthermore, the included studies are characterized by a high risk of bias, which further limits the ability to draw firm conclusions.

As of today, no conclusive evidence supports the effect of LNVA for treating lymphedema. The procedure seems promising, and future research should focus on performing well-conducted prospective clinical trials with systematic measurements of patients undergoing LNVA surgery to evaluate its efficacy fully.

## Conclusion

Current evidence on LNVA for treating lymphedema is limited and of low methodological quality, preventing firm conclusions regarding its efficacy. Although advances in surgical technique and microsurgical technology have renewed interest in LNVA and suggest potential benefits in selected patients, existing studies are non-randomized and subject to significant bias. Well-designed prospective studies are needed to evaluate the true clinical value of LNVA in lymphedema treatment.

## Appendix Supplementary materials

Image, application 1
