# Supplementary material for: LNVA for treating secondary lymphedema: A systematic review
Source: JPRAS Open. 2026 Aug 3;51:775–83. doi: 10.1016/j.jpra.2026.07.035 (PMC13543071; doi:10.1016/j.jpra.2026.07.035)
Supplement: Supplementary file 1 [file mmc1.docx]

# Appendix

| Search String | |
| --- | --- |
| Embase | (Lymph node to vein anastomosis.mp OR LNVA.mp) AND (exp lymphatic system/or exp lymphedema/ or secondary lymphedema.mp) |
| Medline | (Lymph node to vein anastomosis.mp OR LNVA.mp) AND (exp Lymphedema/ or secondary lymphedema.mp) |
| Cochrane Library | (Lymph node to vein anastomosis OR LNVA) AND (lymphedema) |
| Google Scholar | (lymph node to vein anastomosis OR LNVA) AND (lymphedema) |
| ClinicalTrials.gov | Condition/disease: Lymphedema  Other terms: Secondary Lymphedema  Intervention/treatment: lymp node to vein anastomosis  Study Status: All studies |
| ICTRP | (Lymph node to vein anastomosis OR LNVA) AND lymphedema |

*Table 1a: Overview of the search string for each database.*
